# *In vitro* spermatogenesis: In search of fully defined conditions

**DOI:** 10.3389/fcell.2023.1106111

**Published:** 2023-02-24

**Authors:** A. Yu. Kulibin, E. A. Malolina

**Affiliations:** Koltzov Institute of Developmental Biology of the Russian Academy of Sciences, Moscow, Russia

**Keywords:** *in vitro* meiosis, germ cell, cell culture, organ culture, 3D-culture, haploid cells

## Abstract

A complete reconstruction of spermatogenesis *in vitro* under fully defined conditions still has not been achieved. However, many techniques have been proposed to get closer to that aim. Here we review the current progress in the field. At first, we describe the most successful technique, the organ culture method, which allows to produce functional haploid cells. However, this method is based on the culturing of intact testis tissue with unknown factors acting inside it. Then we discuss different types of 3D-cultures where specific testicular cell populations may be aggregated and the impact of each cell population may be examined. Unfortunately, germ cell development does not proceed further than the pachytene stage of meiosis there, with rare exceptions. Finally, we describe recent studies that focus on germ cells in a conventional adherent cell culture. Such studies thoroughly examine issues with *in vitro* meiosis and provide insight into the mechanisms of meiotic initiation.

## Introduction

Spermatogenesis is the process of male germ cell differentiation to spermatozoa (reviewed in Jan et al. (2012)). It begins with the differentiation of undifferentiated spermatogonial cells. The differentiated spermatogonia then enter meiosis, the central and the most important part of spermatogenesis. Germ cells, now called spermatocytes, undergo meiotic recombination and reductive cell division, which results in the formation of haploid round spermatids. They can already be used for *in vitro* fertilization (Ogura et al., 1994). Finally, round spermatids are transformed into elongated spermatids and then into spermatozoa in the process of spermiogenesis. In Mammals, spermatogenesis takes place inside the seminiferous tubules of testes and is supported by gonadal somatic cells, mainly Sertoli cells, which are in direct contact with germ cells, but also Leydig cells, peritubular myoid cells, and others (Jan et al., 2012).

Multiple factors, such as growth factors, cytokines, nutrients, and hormones, might affect spermatogenesis, and some of them, like hormones, act indirectly, through testicular somatic cells. Which factors and conditions turn out to be critical for spermatogenesis is not completely understood. So, it is still not possible to reproduce the whole process of spermatogenesis under fully defined conditions *in vitro*. Achieving this goal would be of great scientific interest *per se* because it would mean that all the basic principles of male germ cell development were elucidated. *In vitro* spermatogenesis could in its turn become a model system for studying processes that were difficult or impossible to examine *in vivo*, for example, human gametogenesis. Such a model system would also be convenient for studying the mechanisms of testicular disorders, drug testing, and assessing effects of toxic compounds on testicular cells. And finally, *in vitro* spermatogenesis could be used to treat male infertility but only after comprehensive analysis of human haploid cells generated *in vitro* as well as after careful examination of offspring produced from *in vitro* differentiated haploid cells of model animals.

There are many studies reporting the reconstruction of some stages of the spermatogenic process *in vitro* and production of meiotic and haploid cells. However, in this review, we will discuss only those studies that thoroughly examined meiosis, which is a main challenge for *in vitro* gametogenesis (Handel et al., 2014), and/or obtained offspring from *in vitro* generated gametes, which is the ultimate evidence of correct gametogenesis (Handel et al., 2014). Some studies not meeting these criteria are mentioned in the review because they developed novel approaches which could, in our opinion, be useful in the field of *in vitro* spermatogenesis.

The review will focus on *in vitro* spermatogenesis *per se* and will not describe the generation of undifferentiated germ cells from pluripotent stem cells. Let us just mention that functional primordial germ cell-like cells (PGCLCs) were produced from embryonic stem cells (ESCs) and induced pluripotent stem cells (Toyooka et al., 2003; Hayashi et al., 2011), and that spermatogonium-like cells were in turn derived from PGCLCs after their aggregation and subsequent culturing with fetal testicular cells (Ishikura et al., 2021).

## Spermatogenesis in an organ culture

**FIGURE 1:**
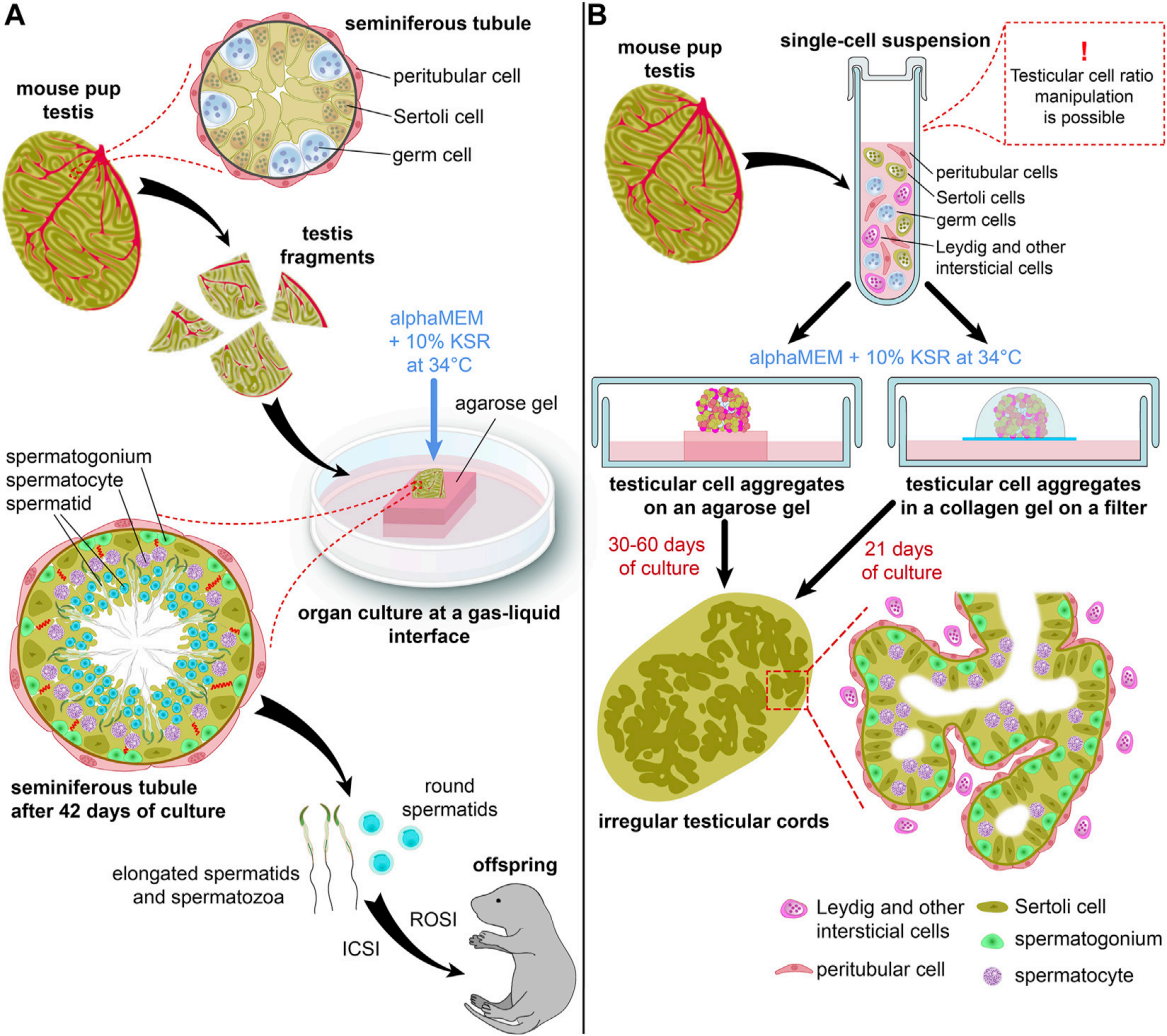
Schematic representations of an organ culture method **(A)** and a 3D-culture method **(B)** for *in vitro* spermatogenesis. **(B)** shows two types of 3D-culture producing more advanced germ cells.

An organ culture method, which maintains tissue fragments or whole organs *in vitro* with minimum disturbance of tissue architecture, has been used for male germ cell differentiation since the 1930s. It allowed to reproduce *in vitro* the development of rat germ cells from spermatogonia to pachytene spermatocytes but not beyond in the 1960s (reviewed in Steinberger and Steinberger, 1970). In 2010 (Gohbara et al., 2010) and 2011 (Sato et al., 2011a; Sato et al., 2011), three studies from one research team achieved a huge progress in that technique. The authors modified a gas-liquid interface method developed earlier (Trowell, 1959) and cultured testis tissue fragments from immature mice on the surface of agarose gel half-soaked in a medium (Figure 1A). This technique allowed easy access to oxygen from the air and to the medium nutrients from agarose gel. Different culture conditions were tested, and maintaining the tissue in alphaMEM medium at 34°C was found optimal for spermatogenesis (Gohbara et al., 2010). But the main achievement was the replacement of fetal bovine serum with KSR supplement (Sato et al., 2011).

The authors used two lines of transgenic mice: Acr (acrosin)-GFP and Gsg2 (haspin)-GFP, to monitor the progression of spermatogenesis. The first one expressed GFP in male germ cells from the mid-pachytene stage of meiosis onwards, while the second one started to express GFP during meiotic cell divisions (Gohbara et al., 2010). Testicular fragments cultured with 10% KSR showed dramatically higher levels of Acr-GFP and Haspin-GFP expression than those cultured with 10% FBS (Sato et al., 2011). Also, KSR prolonged the GFP expression, and spermatogenesis was maintained *in vitro* over 2 months. Staining for SYCP1 and SYCP3, components of the synaptonemal complex (Dobson et al., 1994), confirmed meiosis progression. Analysis of DNA content by flow cytometry and Acr-GFP expression in acrosomes indicated the formation of spermatids. Even flagellated sperm was observed in some samples. Moreover, fertile offspring was obtained both from the round spermatids and the sperm by ROSI (round spermatid injection) and ICSI (intracytoplasmic sperm injection) (Sato et al., 2011) (Figure 1A).

In the third study (Sato et al., 2011a), the organ culture method was combined with the transplantation of spermatogonial stem cells (SSCs), which was developed earlier to induce spermatogenesis from donor germ cells *in vivo* (Brinster and Avarbock, 1994; Brinster and Zimmermann, 1994). The authors injected germ cells into the seminiferous tubules of a host testis, and the fragments of the host testis were then cultured with 10% KSR in agarose gel at a gas-liquid interface. As in the previous studies, complete spermatogenesis from transplanted cells was shown, and haploid cells were obtained that were used for successful artificial fertilization (Sato et al., 2011a). Recently, this technique was applied to reproduce *in vitro* the whole male germ cell development: from mouse ESCs through the stages of primordial germ cells and spermatogonia and to elongated spermatids (Ishikura et al., 2021). An organ culture was used at the last step: specifically, for the differentiation of spermatogonia and spermatogenesis.

The same research team applied the organ culture method to a wide range of situations. They demonstrated offspring production with haploid cells grown from cryopreserved testis tissue of neonatal mice (Yokonishi et al., 2014). They reported that this approach could also be employed for fetal testes (Kojima et al., 2016) and adult testes (Sato et al., 2015), although with lower efficiency than that of neonatal testes. The organ cultures exhibited round and elongating spermatids in both cases, but offspring was not obtained. They increased the efficiency of spermatogenesis induction *in vitro* by constructing microfluidic devices which flattened testis tissue, imitated the microvascular flow, and thereby promoted an even distribution of nutrients and oxygen throughout a testis specimen (Komeya et al., 2016; 2017; Yamanaka et al., 2018). These devices also allowed to maintain spermatogenesis *in vitro* for extended periods of time (Komeya et al., 2016; Yamanaka et al., 2018). Even a simple flattening of testis tissue with an oxygen permeable chip substantially improved cell viability and support growth of cultured tissue (Kojima et al., 2018).

Other researchers started routinely applying the organ culture method to studying male germ cell development on mice (Arkoun et al., 2015; Isoler-Alcaraz et al., 2017 etc.). Organ cultures of testis tissue from rat pups were also established (Reda et al., 2016; Matsumura et al., 2021; Nakamura and Sloper, 2021; Saulnier et al., 2023). However, rat *in vitro* spermatogenesis proceeded only up to round spermatids, and the numbers of haploid cells were low (Reda et al., 2016), even after additional modifications of culture conditions and a medium (Matsumura et al., 2021; Nakamura and Sloper, 2021; Saulnier et al., 2023). In addition, the ability of these cells to generate offspring with ROSI was not yet tested.

*In vitro* reconstruction of human spermatogenesis faces obvious technical and ethical obstacles including the long duration of human spermatogenesis as well as the impossibility to obtain the starting material from healthy donors not receiving hormone therapy and to confirm the competency of *in vitro* generated human haploid cells to produce offspring. Nevertheless, one study (Yuan et al., 2020) utilized fetal gonads from aborted fetuses and reported an organ culture of human testis tissue with spermatogenesis proceeding up to round spermatids. Testicular maturation and germ cell development were clearly accelerated in this study. However, the authors demonstrated correct distribution of some meiotic markers, such as SYCP3, MLH1, and γH2AX, comprehensively characterized generated spermatids, and confirmed their ability to fertilize oocytes and support early embryo development (Yuan et al., 2020).

The success of the organ culture method at least on mice, could be explained by the fact that tissue architecture and close interactions between germ and somatic cells remained mostly intact. That allowed to use an organ culture of testis tissue as a model system for examining basic factors and conditions that are vital for spermatogenesis and relatively independent of cell-cell interactions, such as temperature, nutrients, hormones. The detrimental effect of temperature higher than normal scrotal temperature, specifically 35°C or higher, for rat *in vitro* spermatogenesis was demonstrated in early studies (Steinberger and Steinberger, 1970), and, as mentioned above, 34°C was found the best for mouse *in vitro* spermatogenesis (Gohbara et al., 2010).

Much effort has been made to develop an optimal medium for *in vitro* spermatogenesis. In one of the first studies on the organ culture method (Sato et al., 2011), the authors reported that FBS did not contain factors suppressing spermatogenesis and that lipid-rich bovine serum albumin (AlbuMAX, 40 mg/ml), the main component of KSR, was as effective in supporting spermatogenesis as KSR. However, neither AlbuMAX nor KSR was defined chemically. So, further efforts were focused on identifying key molecules that were essential for male germ cell development *in vitro* and on creating a chemically defined medium (Sanjo et al., 2018; Sanjo et al., 2020).

It was shown that retinoic acid (RA, present in AlbuMAX) and retinol, well-known inductors of spermatogonial differentiation (reviewed in Gewiss et al., 2021), as well as lipids from AlbuMAX, such as free fatty acids (FFA), cholesterol, phosphatidylcholine, and sphingomyelin, promoted meiotic initiation (Sanjo et al., 2018). Testosterone, LH, FSH, and triiodothyronine (T3) were present in AlbuMAX, albeit the first three of them at very low concentrations. The combination of these hormones strongly enhanced Acr-GFP expression (Sanjo et al., 2018), with T3 and testosterone showing the greatest effect (Sanjo et al., 2020). Antioxidant vitamins, such as α-tocopherol (present in AlbuMAX), ascorbic acid, and glutathione, dramatically increased Acr-GFP expression, which meant that the number of germ cells reaching the pachytene stage of meiosis increased (Sanjo et al., 2020). Addition of the lysophospholipids from AlbuMAX further increased Acr-GFP expression up to the levels examined in specimens cultured with AlbuMAX (Sanjo et al., 2020). However, spermatid formation was still disrupted in the chemically defined medium, which meant that not all critical factors in AlbuMAX were identified (Sanjo et al., 2020).

Nevertheless, the molecules promoting spermatogenesis that have already been discovered, such as hormones, antioxidants, and lysophospholipids, have been recently used as supplements for the AlbuMAX medium to increase the effectiveness of spermatogenesis in an organ culture (Ishikura et al., 2021; Matsumura et al., 2021). Moreover, many basic factors and culture conditions identified using an organ culture of testis tissue have been applied to cultures of testicular cells.

## Spermatogenesis in a 3D-culture

A further step to more defined conditions for *in vitro* spermatogenesis would be a three-dimensional (3D) culture system of testicular somatic cells and germ cells. 3D-culture allows cells to interact with each other and with extracellular matrix in three dimensions and to recapitulate tissue architecture. A 3D-culture of testicular cells with complete spermatogenesis could provide valuable information about critical factors in testicular microenvironment affecting germ cell development and about the role of different populations of somatic cells in spermatogenesis.

The most successful attempt to initiate spermatogenesis in a 3D culture was made in 2013 (Yokonishi et al., 2013) by the same research team which achieved full spermatogenesis in an organ culture (Sato et al., 2011). It had been reported previously that dissociated immature testicular cells developed structures resembling testis cords and even seminiferous tubules after being grafted underneath a kidney capsule (Dufour et al., 2002) and subcutaneously (Gassei et al., 2006). It was also shown that complete spermatogenesis could be induced in such conditions, and normal offspring could be produced from the ectopically generated round spermatids by ROSI (Kita et al., 2007; Matoba and Ogura, 2011).

In 2013 (Yokonishi et al., 2013), the researchers used similar techniques, but they cultured testicular cell aggregates according to the organ culture method on agarose gel in alphaMEM with 10% KSR (Sato et al., 2011), instead of grafting them (Figure 1B). Testicular somatic cells formed irregular tubular structures resembling seminiferous tubules. Those structures contained germ cells, which differentiated up to pachytene spermatocytes, according to Acr-GFP expression and staining for SYCP1. Haploid cells were also probably formed there, but in small numbers (Yokonishi et al., 2013).

Another 3D culture system was proposed in 2014 (Zhang et al., 2014) with testicular cells placed inside a collagen matrix and cultured on a floating membrane filter at a gas-liquid interface in a medium with 10% KSR (Figure 1B). Seminiferous tubule-like structures were formed by Sertoli cells which gradually differentiated, and spermatogenesis was initiated in the tubules up to the spermatocyte stage, which was confirmed by SYCP3 staining (Zhang et al., 2014). Other methods, such as a soft agar culture system (Stukenborg et al., 2009; Abu Elhija et al., 2012), culturing in Matrigel (Gassei et al., 2010) and in a multilayer gradient system based on Matrigel (Alves-Lopes et al., 2018), as well as generation of 3D testicular organoids in hanging transwell inserts (Baert et al., 2017) and microwells (Sakib et al., 2019) were also reported. However, they were not employed for germ cell development (Gassei et al., 2010), or only supported spermatogonial cells (Baert et al., 2017; Alves-Lopes et al., 2018; Sakib et al., 2019), or else there was not enough evidence of meiosis progression and functionality of haploid cells (Stukenborg et al., 2009; Abu Elhija et al., 2012). 3D printed hydrogel scaffolds were also developed for culturing immature testicular cells; however, the germ cell development did not proceed further than the zygotene stage of meiosis (Richer et al., 2021).

So, the 3D methods described above seem to be far less effective than the organ culture method: most significantly, no functional spermatids were produced when using them. In (Yokonishi et al., 2013) some reasons for that were suggested: specifically, the unbalanced composition of somatic cell populations and poor incorporation of spermatogonia into the reconstructed tubular structures, in comparison with the high capacity of primordial germ cells to aggregate with Sertoli cell precursors in the embryo. Also, as far as we know, the effect of hormones and other key molecules identified in (Sanjo et al., 2018; Sanjo et al., 2020), which could promote spermatogenesis, was not examined in 3D-cultures.

## Spermatogenesis in a “SEMI-3D” culture

**FIGURE 2:**
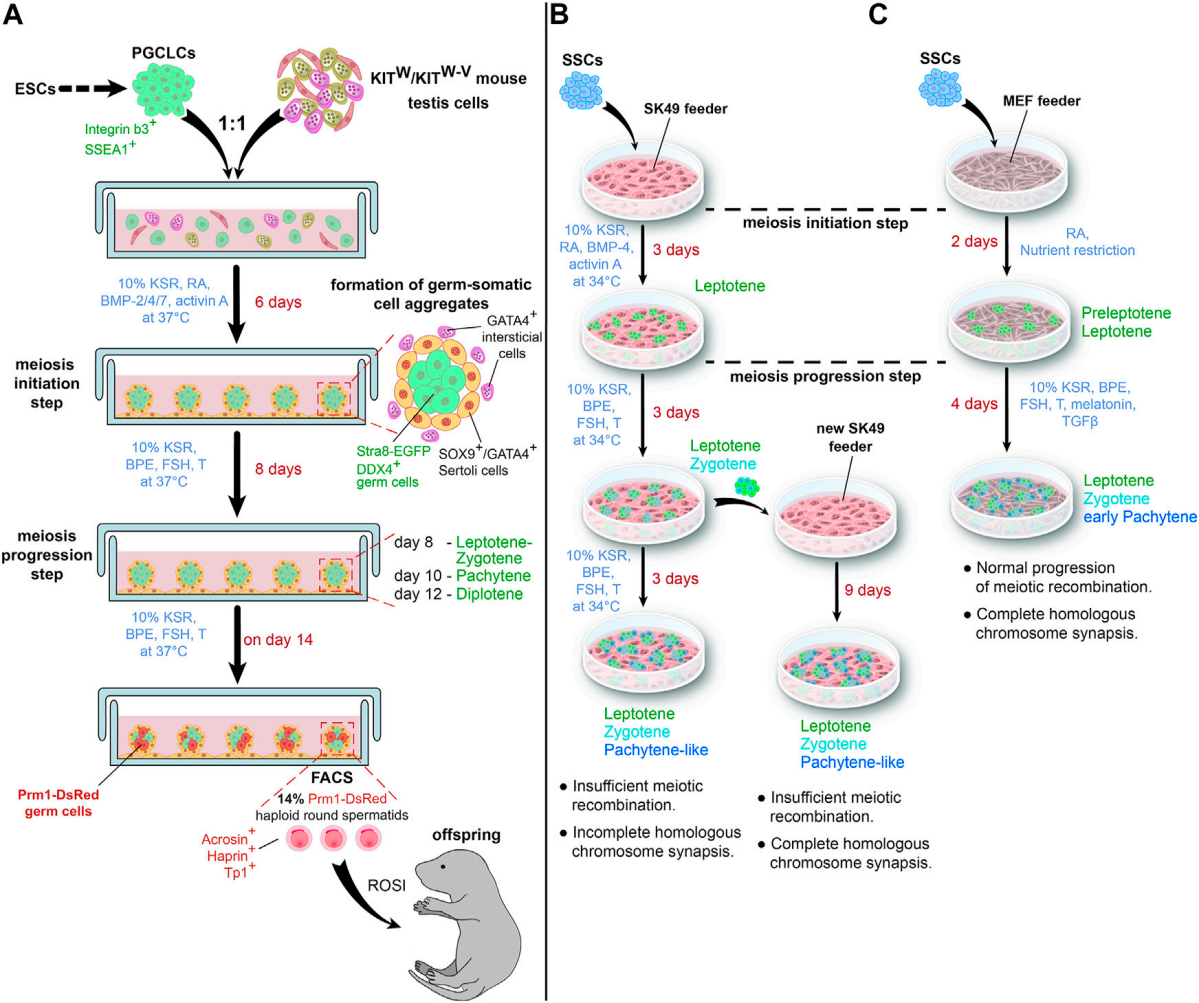
Schematic representations of a “semi-3D” culture method **(A)** and an adherent cell culture method **(B,C)** for *in vitro* spermatogenesis. **(C)** illustrates the impact of a nutrient restriction step on *in vitro* meiosis. T—testosterone.

In 2016 Zhou et al. (2016) partly addressed those issues when they reported a complete meiosis of male germ cells in culture conditions that can be called “semi-3D”. They used PGCLCs derived from ESCs according to a protocol developed by Hayashi et al. (2011). PGCLCs were mixed with testicular cells isolated from germ cell-deficient KIT^W^/KIT^W−V^ pups at a ratio of 1:1. The two types of cells were co-cultured in a medium supplemented with 10% KSR, RA, BMP-2/4/7, and activin A for 6 days (Figure 2A). The BMPs and activin A promoted proliferation and also upregulated the expression of *Ddx4*, a germ cell marker (Fujiwara et al., 1994), and *Nanos3*, a marker of undifferentiated germ cells (Tsuda et al., 2003). It seems that these factors promoted the conversion of PGCLCs into the spermatogonial state. RA activated the expression of *Stra8* and *Rec8*, which are essential for meiosis initiation (Anderson et al., 2008; Koubova et al., 2014). At the same time, testicular somatic cells aggregated with germ cells, forming 3D structures and reconstituting the microenvironment of seminiferous tubules (Figure 1B). From day 7 to day 14 the co-culture was maintained in the medium containing 10% KSR, bovine pituitary extract (BPE), FSH, and testosterone (Zhou et al., 2016).

Analysis of germ cell nuclei on day 8 showed multiple foci of γH2AX, a phosphorylated histone, which marks DNA double-strand breaks (DSBs, Kinner et al., 2008); SPO11, a DNA topoisomerase, which generates meiotic DSBs (Romanienko and Camerini-Otero, 2000); and RAD51, a recombinase participating in DSB repair (Bishop, 2012). Transcripts of DMC1, a meiosis-specific recombinase repairing DSBs (Bishop et al., 1992; Yoshida et al., 1998), increased dramatically on days 6–10 and decreased by day 14. By day 10, γH2AX disappeared from the autosomes and remained only in the XY body. All these facts indicated the formation and subsequent repair of meiotic DSBs *in vitro* (Zhou et al., 2016).

Co-staining for SYCP1, a component of the transverse filaments of the synaptonemal complex (Meuwissen et al., 1992), and SYCP3 from the lateral elements of the complex (Lammers et al., 1994; Yuan et al., 2000), allowed to stage the differentiating germ cells and to demonstrate the progression and completion of the synapsis. The process was quite effective, as more than 60% of the spermatocytes were at the pachytene stage at day 10 and more than 50% reached the diplotene stage on day 12. Proper formation of bivalents in metaphase I was confirmed by karyotyping (Zhou et al., 2016).

By day 14, the markers of haploid cells, Prm1, acrosin, haprin, and Tp1, became upregulated. FACS analysis revealed 14% of cells with 1C DNA. Cells from Prm1-DsRed transgenic mice were used for *in vitro* spermatogenesis to sort out PRM1-positive cells from the culture. The yield of such cells was about 2 × 10^4^ cells per well, which was quite high considering that the initial number of PGCLCs per well was 5 × 10^4^. Most of PRM1-positive cells were haploid and positively stained for acrosome marker peanut agglutinin. Finally, the authors performed ICSI with the sorted cells and obtained fertile offspring (Zhou et al., 2016).

It is important to note that all three supplements, specifically BPE, FSH, and testosterone, were indispensable for the production of haploid cells (Zhou et al., 2016). Although the authors did not stain the co-culture for the receptors for FSH, testosterone, and LH, we can assume their presence in the somatic cells because testicular cells were freshly isolated and cultured in semi 3D-conditions, which were closer to an *in vivo* situation than an adherent cell culture.

This study (Zhou et al., 2016) has demonstrated the possibility of complete spermatogenesis in 3D aggregates of germ and somatic testicular cells *in vitro* and the importance of hormone supplementation for spermatogenesis in a 3D-culture. So, the unbalanced composition of cell populations in aggregated testicular structures (Yokonishi et al., 2013) may not be an obstacle. However, following studies utilizing this technique are absent, so the reproducibility of this approach is not clear.

## Spermatogenesis in an adherent cell culture

The next step would be to achieve spermatogenesis in an adherent cell culture, where germ cells differentiated on a layer of feeder cells. That is a challenging task, because factors in testicular microenvironment which are critical for spermatogenesis have not yet been identified. There are few studies investigating meiosis of testicular germ cells in such a culture.

Lei et al. (2020); Lei et al. (2021) conducted two studies in 2020 and 2021 where they used an immortalized Sertoli cell line SK49 as a feeder layer to support SSC differentiation. The spermatogonial stem cell line was isolated from neonatal DBA/2J male mice and maintained as reported by Kanatsu-Shinohara et al. (2003). At first, spermatogonial differentiation and meiosis initiation were induced by replacing the medium for GSCs with a medium containing 10% KSR, RA, BMP4, and activin A. After 3 days, when first leptotene cells appeared, the medium was changed again to a medium for meiosis progression with 10% KSR, BPE, FSH, and testosterone. The culture was maintained for the next 6 days (Lei et al., 2020) (Figure 2B).

The authors thoroughly compared the *in vitro*-formed spermatocytes with their *in vivo* counterparts. The staining for SYCP3, the main protein of the lateral elements of the synaptonemal complex (Lammers et al., 1994; Yuan et al., 2000), was used to identify meiosis stages. It was found that the phosphorylated histone γH2AX and recombinase RAD51 were still present on autosomes during the *in vitro* pachytene stage, indicating that DSB repair was not completed. MLH1, an endonuclease participating in crossing over (Hunter and Borts, 1997), was not observed at the pachytene stage. And bivalents were not formed *in vitro* (Lei et al., 2020).

A transfer of differentiating germ cells after 3 days of meiosis progression to a fresh feeder led to some improvements (Lei et al., 2021). Specifically, at the pachytene stage, staining for γH2AX disappeared from autosomes and remained only in the XY body, and MLH1 foci appeared in chromosomes. However, RAD51 was still present *in vitro* pachytene spermatocytes, and the number of MLH1 foci was quite low (Figure 2B).

In this study (Lei et al., 2020), the authors also analyzed the distribution of other meiotic proteins. They demonstrated the normal formation of the synaptonemal complex *in vitro*, as SYCP3 was fully co-localized with SYCP1 at the pachytene stage. Also, HORMAD1, a protein marking unsynapsed chromosomes (Wojtasz et al., 2009), disappeared from autosomes at the pachytene stage as it did *in vivo*.

On the whole, these two studies (Lei et al., 2020; Lei et al., 2021) demonstrated the completion of the meiotic synapsis in a conventional adherent cell culture, but not the meiotic recombination. In addition, only few pachytene spermatocytes were generated and most of the germ cells were at the leptotene and zygotene stages during the whole culture time.

Recently, Zhang et al. (2021) proposed an interesting hypothesis that RA was not sufficient to initiate meiosis in mammals, and that nutrient restriction, which is a signal to induce meiosis in yeasts, was also necessary for that purpose. The authors supposed that specific conditions with low concentrations of some nutrients were created inside seminiferous tubules to support meiosis in male germ cells *in vivo*.

To test their hypothesis, Zhang et al. (2021) differentiated mouse SSCs on a feeder of mouse embryonic fibroblasts (MEFs). They incubated the cells for 2 days with RA in SSC medium diluted in a ratio of 1:9 with Earle’s Balanced Salt Solution and showed loss of SSC marker PLZF (Costoya et al., 2004) and rise of meiotic transcripts *Spo11*, *Dmc1*, and *Sycp3*. Staining for γH2AX and DMC1, a meiosis-specific recombinase, confirmed the formation of meiotic DSBs (Figure 2C).

On day 3, the cells were transferred to a medium for meiotic progression containing 10% KSR, BPE, FSH, testosterone, melatonin, and transforming growth factor (TGF)-β; the cells were then cultured for another 4 days. Further analysis of DSBs showed that MEIOB and SPATA22, which cooperatively bind to meiotic DSBs to promote recombination (Xu et al., 2017), were present in the nuclei of differentiating germ cells, as well as recombinase RAD51. Moreover, the numbers of MEIOB, SPATA22, DMC1, and RAD51 foci were similar to *in vivo* meiosis. The authors also demonstrated a proper formation of the synaptonemal complex by co-staining the cells with SYCP3 and SYCP1. By day 6, the germ cells had reached the early pachytene stage. In contrast, treatment with RA alone resulted in the arrest of most cells at the preleptotene stage (Zhang et al., 2021).

So, Zhang et al. (2021) have demonstrated that meiosis could be induced in male germ cells in the conventional culture even without gonadal somatic cells and that nutrient restriction was necessary for that.

These three studies (Lei et al., 2020; Lei et al., 2021; Zhang et al., 2021) have achieved certain progress in the reconstruction of the meiosis process in the conventional culture. However, development of male germ cells beyond the pachytene stage still remains a challenge. It seems that culture conditions for meiosis progression were suboptimal. Indeed, FSH and testosterone, which are two crucial hormones for spermatogenesis (reviewed in O'Shaughnessy, 2014), act on germ cells indirectly and mostly through Sertoli cells. Germ cells do not have receptors for these hormones, and there is no evidence that Sertoli cell line SK49 and MEFs have the functional receptors too. Future studies are needed to investigate this issue.

## Perspectives of *in vitro* spermatogenesis

Although many achievements have been made in the field of *in vitro* spermatogenesis, some key issues remain to be solved. The most successful approach is an organ culture of mouse testis tissue. However, the number of haploid cells in this culture is still low, as compared to *in vivo* testes (Abe et al., 2020), and the beginning of spermatogenesis is delayed (Isoler-Alcaraz et al., 2017). Microarray analysis and flow cytometry reveal that spermatogenesis in an organ culture is partially arrested at the stage of meiosis initiation (Abe et al., 2020). It seems that the same issue persists in other types of cultures trying to reproduce germ cell development *in vitro*. Overcoming this problem may increase the efficiency of spermatogenesis in all culture systems.

Another issue is connected with the need to use somatic cells. The conception of organoids widely used for other organs and tissues is hardly applicable to the testis. Organoids are 3D-structures that recapitulate basic tissue architectures and functionalities and that develop from stem cells or progenitors (Rossi et al., 2018). However, SSCs are not able to self-organize into seminiferous epithelium without somatic cells, specifically supporting Sertoli cells and possibly interstitial cells. Whereas at least some types of interstitial cells have stem cells or progenitors (Zhao et al., 2021; Ademi et al., 2022), Sertoli cells do not. So, the choice is to use immature testis tissue (Sato et al., 2011; Ishikura et al., 2021 etc.) or immature Sertoli cells which are not stem or progenitor cells but are capable to proliferate and to self-organize into seminiferous tubules (Yokonishi et al., 2013; Zhou et al., 2016). Another possibility is to differentiate Sertoli cells from pluripotent stem cells, and there are some advances in this field (Knarston et al., 2020). However, the correctness of Sertoli cell maturation *in vitro* is unclear, which may be the reason for the low efficiency of *in vitro* spermatogenesis. This suggestion is also true for other organs, as organoids derived from pluripotent stem cells or fetal progenitors are mainly used to study organogenesis and rarely reach an adult tissue stage, whereas organoids derived from adult cells are considered to be the best for the reproduction of adult tissue functions (Rossi et al., 2018). The solution to this issue would be the development of a protocol for Sertoli cell maturation *in vitro* or a technique for efficient and correct meiosis of male germ cells without support of somatic cells.
